# Rib cage contributions to inspiratory capacity in patients with cervical spinal cord injury

**DOI:** 10.1016/j.crphys.2024.100127

**Published:** 2024-05-17

**Authors:** Ryo Yoshida, Kenta Kawamura, Yukako Setaka, Hyunjae Woo, Nobuhisa Ishii, Masafumi Mizukami, Hirotaka Mutsuzaki, Kazuhide Tomita

**Affiliations:** a Graduate School of Health Science, Ibaraki Prefectural University of Health Sciences, Japan; b Department of Physical Therapy, Ibaraki Prefectural University of Health Sciences, Ibaraki, Japan; c Ibaraki Prefectural Central Hospital, Ibaraki, Japan

**Keywords:** Rib cage motion, Inspiratory capacity, Cervical spinal cord injury, Respiratory inductance plethysmography

## Abstract

**Background:**

Cervical spinal cord injury (CSI) often leads to impaired respiratory function, affecting the overall well-being of patients. This study aimed to investigate the influence of rib cage motion on inspiratory capacity in CSI patients.

**Methods:**

We conducted a study with 11 CSI patients, utilising respiratory inductance plethysmography (RIP). We measured ventilatory volume by spirometry concurrently with RIP. Participants were instructed to perform maximal inspiratory efforts. Inspiratory capacity (IC) was calculated from spirometry waveforms. We converted the respiratory waveforms of the chest and abdomen into inspiratory volume measured by a spirometer. The inspiratory volume measured by the chest sensor was defined as V_RIP-rib cage_ (V_RIP-rc_), and the inspiratory volume measured by the abdominal sensor was defined as V_RIP-abdomen_ (V_RIP-ab_). Subsequently, the relationships of IC with V_RIP-rc_ and V_RIPab_ were assessed.

**Results:**

The mean IC was 1.828 ± 0.459 L, with the mean V_RIP-rc_ at 1.343 ± 0.568 L and the mean V_RIP-ab_ at 0.485 ± 0.427 L. A significant correlation was observed between IC and V_RIP-rc_ (r = 0.67, p = 0.02), indicating that rib cage motion significantly influences IC in CSI patients.

**Conclusion:**

This study highlights the importance of rib cage motion in assessing inspiratory capacity in patients with CSI.

## Highlights

• CSI impacts respiratory function, affecting patient well-being.
• RIP and spirometry reveal significant correlation in CSI patients.
• Rib cage motion crucial in assessing inspiratory capacity in CSI.

## 1 Introduction

The estimated incidence of cervical spinal cord injury (CSI), excluding Frankel E cases, was 49 per million in Japan 2018. The overall rate of cervical cord injuries was 88.1% among study subjects. Among patients with spinal cord injury, injuries to the cervical spinal cord were the most common (Miyakoshi et al., 2021). Patients with CSI experience respiratory compromise due to respiratory muscle paralysis (Linn et al., 2001; Liaw et al., 2000), which is reported to cause increased dyspnoea in daily life and decreased health-related quality of life (QOL) (Jain et al., 2007). Additionally, respiratory complications are the major cause of mortality in patients with CSI (Frankel et al., 1998). Coughing is important in preventing respiratory complications, and the ability to cough depends on the strength of the inspiratory muscles (Kang et al., 2006). Therefore, we emphasise the importance of assessing the inspiratory capacity of patients with CSI to prognosticate their survival and QOL. Inspiration is generally evaluated using spirometry. However, since spirometry cannot discriminate ventilatory changes specifically attributed to respiratory muscles such as the diaphragm and intercostal muscles, an alternative approach is required to analyse the recovery of respiratory function after a cervical spinal cord injury.

In clinical practice, the assessment of rib cage motion serves as an essential tool for evaluating the respiratory status of individuals. Studies have demonstrated a correlation between rib cage motion, respiratory muscle strength, and VC, with individuals exhibiting greater rib cage motion tending to have higher values for both PImax and VC (Lanza, et al., 2013). Conversely, it has been reported that the diaphragm expands the abdomen during inspiration (Grassino, et al., 1978). Hence, visualising these motions serves as a simple index of inspiration. Respiration involves the synchronised motion of two compartments: the rib cage and abdomen (Konno and Mead, 1967). The respiratory muscles execute the expansion and contraction of these two compartments, which are perceived as the motion of the rib cage and abdomen. Respiratory inductance plethysmography (RIP) is employed for the assessment of rib cage and abdomen motion (DiMarco and Kowalski, 2015). RIP quantifies ventilatory volume based on changes in the rib cage and abdominal volumes during respiratory cycles. These volumetric parameters are measured separately, allowing the assessment of inspiratory function by analysing the motions of both the rib cage and abdomen. RIP is also employed for the assessment of patients with CSI. In a previous study, we investigated rib cage motion in three patients with CSI and found an association between rib cage motion and increased inspiratory capacity (IC). Furthermore, our findings indicated a proportional reduction in rib cage motion with the severity of inspiratory muscle paralysis in CSI patients. (Yoshida et al., 2021). However, limited literature exists on rib cage motion in patients with CSI. Furthermore, it remains unclear which of the rib cage and abdominal motion is more suitable for evaluating inspiratory function.

This study aimed to investigate the relationship between IC, rib cage, and abdominal motion in patients with CSI using RIP.

## 2 Materials and methods

### 2.1 Participants

Eleven people registered for the study, as shown in Table 1, Table 2. The inclusion criteria included being diagnosed with CSI and consenting to participate in the research. The exclusion criteria included having difficulty following instructions and having respiratory diseases. No participants were excluded by the selection criteria. This study has been approved by the Ethics Committee of Ibaraki Prefectural University of Health Sciences (approval number: 830). All participants provided informed consent.

**Table 1:** Physical characteristics of the subjects.

		Mean value
Age	[years]	59.8 ± 13.5
Sex	[Male:Female]	11:0
Height	[cm]	164.4 ± 5.7
Weight	[kg]	65.3 ± 8.8
BMI	[kg/m^2^]	24.1 ± 2.8
VC	[L]	2.82 ± 0.87
%VC	[%]	80.0 ± 23.7

Abbreviations: BMI, body mass index; VC, vital capacity; %VC, vital capacity as percent of predicted.

Values in this table are expressed as mean ± standard deviation.

**Table 2:** Detailed information of the subject.

Case	Age [years]	AIS	Neurological damage level		ASIA motor score		Time after injury [years, months]
			Sensory	Motor	Upper limbs	Lower limbs	
1	49	A	C1	C8	45	0	23 y 1 m
2	42	D	C5	C5	44	40	15 y 2 m
3	62	D	C5	C5	28	40	4 m
4	37	D	C2	L2	50	42	4 m
5	71	D	C4	C4	37	50	1 m
6	81	A	C5	C5	10	0	17 y 1 m
7	69	C	C4	C4	24	5	9 m
8	56	D	C4	C4	36	32	1 y 9 m
9	66	D	C4	C6	44	46	4 y 10 m
10	71	D	C5	C7	41	39	19 y 7 m
11	54	D	C2	C4	26	50	4 m

Abbreviations: AIS, American Spinal Injury Association Impairment Scale; ASIA, American Spinal Injury Association.

### 2.2 Experimental equipment and procedure

The thoracoabdominal motion associated with breathing was measured using a RIP device (Respitrace, AMI, U.S.A.). The measurements were performed with the patients in the supine position, with band-shaped sensors (Respibands, AMI, U.S.A.) placed on two areas: the rib cage and abdomen. The sensors were placed on the xiphoid process on the rib cage and directly above the navel on the abdomen. Respitrace analogue signals were converted to digital via an analogue-to-digital (A/D) converter (PowerLab/16/35, ADInstruments, Australia). Ventilatory volume was measured using spirometry concurrently with the measurement of rib cage motion using RIP. Spirometry was recorded after expansion with a respiratory amplifier (ML141 Spirometer, ADInstruments, Australia) via a respiratory resistance tube (MLT300L Respiratory Flow Head 300 L, ADInstruments, Australia) attached to a facemask. Respiratory flow signals were recorded by synchronising the sampling frequency at 1 kHz with an application software for time series analysis on the computer (LabChart 8.0, ADInstruments, Australia).

For the measurement, after respiration at rest for 30 s, the patients were verbally instructed to repeat maximal inspiration three times using the words ‘take a deep breath to expand the chest’. Before the measurement, the subjects practiced enough maximal inspiration and carefully measured IC.

The RIP data with the highest ventilation among the three maximal inspirations were analysed. IC was first calculated from the waveforms measured by spirometry. Subsequently, the respiratory waveforms (mV) of the rib cage and abdomen from the end-expiratory position to the maximal inspiratory position were measured with a respitrace. Then, the respiratory waveforms (mV) of the chest and abdomen from the end-expiratory position to the maximal inspiratory position were converted into the inspiratory volume (L) measured by a spirometer, which was defined as V_RIP_. In addition, the inspiratory volume measured with a spirometer was defined as Volume Pneumotachograph (V_PN_).

### 2.3 Data and statistical analysis

The rib cage motions associated with breathing were calculated as chest respiratory waveform/sum of the chest and abdominal respiratory waveforms. Rib cage volume (V_RIP-rc_) was calculated by the formula [V_RIP_ × ratio of rib cage], and abdominal volume (V_RIP-ab_) was calculated by the formula [V_RIP_ × ratio of abdomen]. Subsequently, the relationships of IC with V_RIP-rc_ and V_RIP-ab_ were assessed for each case.

Statistical analysis was performed using Pearson's product-moment correlation in the analysis of IC and V_RIP-rc_, and Spearman's rank correlation in the analysis of IC and V_RIP-ab_. The data were analysed using IBM SPSS Statistics Ver. 24.0. The significance level was set to 5%.

## 3 Results

**Fig. 1:**
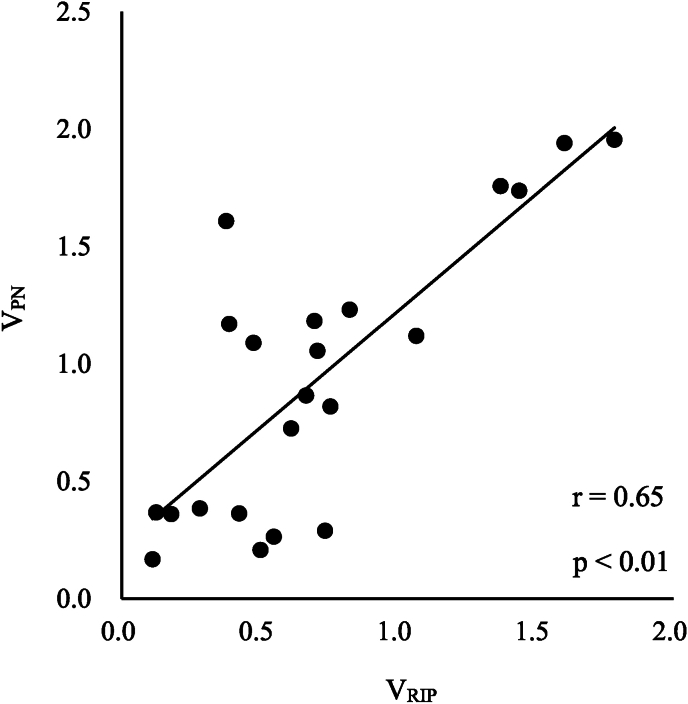
Relationship between V_RIP_ and V_PN_. Two respiratory waveforms were extracted from each subject, and V_RIP_ was calculated. The vertical axis shows the actual ventilation volume measured by the spirometer, and the horizontal axis shows the ventilation volume calculated by RIP after conversion. V_RIP_ and V_PN_ showed a strong correlation, r = 0.65 (p < 0.01). Abbreviations: V_PN_, Volume Pneumotachograph; V_RIP_, respiratory inductance plethysmography volume;

Two respiratory waveforms were extracted from each subject to confirm the measurement accuracy of the RIP device, and V_RIP_ was calculated (Table 3). The vertical axis shows the actual ventilation volume measured by the spirometer, and the horizontal axis shows the ventilation volume calculated by RIP after conversion. A strong correlation was found between V_RIP_ and V_PN_, r = 0.65 (p < 0.01) (Fig. 1).

**Table 3:** V_PN_ and V_RIP_ of the subject.

Case	V_PN_ [L]		V_RIP_ [L]
1	0.819		0.758
	1.119		1.069
2	1.183		0.700
	1.737		1.442
3	0.360		0.180
	1.940		1.607
4	1.056		0.710
	1.758		1.375
5	1.089		0.478
	1.232		0.828
6	0.264		0.552
	0.290		0.738
7	0.363		0.427
	1.955		1.788
8	0.725		0.615
	1.170		0.390
9	0.368		0.126
	0.384		0.284
10	0.208		0.504
	1.609		0.379
11	0.168		0.113
	0.865		0.669
Mean ± SD	0.939 ± 0.594		0.715 ± 0.471

Abbreviations: V_PN_, Volume Pneumotachograph; V_RIP_, respiratory inductance plethysmography volume.

Values in this table are expressed as mean ± SD.

The results of IC, V_RIP-rc_, and V_RIP-ab_ are shown in Table 4. In the rib cage and abdominal motion, the ratios of the rib cage and abdominal motions were 0.734 ± 0.238 and 0.266 ± 0.238, respectively, showing a higher ratio of rib cage motion than abdominal motion. The American Spinal Injury Association Impairment Scale (AIS) is used to evaluate neurological symptoms and diagnose the severity of spinal cord injury. AIS A is very severe and presents with complete paralysis. B, C, and D indicate paresis, and D is less severe. Furthermore, based on the results in Table 4, the average ratio of rib cage motion in AIS A cases and AIS C and D cases was calculated to be 0.452 ± 0.220 in AIS A cases and 0.778 ± 0.201 in AIS C and D cases. The average ratio of rib cage motion was lower in the AIS A case than in AIS C and D cases. The correlation coefficient of IC and V_RIP-rc_ was 0.67, showing a significant correlation (p = 0.02) (Fig. 3, left figure). Contrarily, the correlation coefficient of IC and V_RIP-ab_ was 0.17, showing no significant correlation (Fig. 3, right figure).

**Table 4:** IC, V_RIP-rc_, and V_RIP-ab_ of the subject.

Case	AIS	IC	V_RIP-rc_		V_RIP-ab_	
		[L]	[L]	(Ratio to IC)	[L]	(Ratio to IC)
1	A	1.583	0.429	(0.271)	1.154	(0.729)
2	D	2.167	1.008	(0.465)	1.159	(0.535)
3	D	1.844	0.811	(0.440)	1.033	(0.560)
4	D	2.373	2.068	(0.871)	0.305	(0.129)
5	D	2.162	1.669	(0.772)	0.493	(0.228)
6	A	1.119	0.795	(0.710)	0.324	(0.290)
7	C	2.011	1.645	(0.818)	0.366	(0.182)
8	D	2.174	2.078	(0.956)	0.096	(0.044)
9	D	1.535	1.396	(0.909)	0.139	(0.091)
10	D	2.142	1.891	(0.883)	0.251	(0.117)
11	D	1.002	0.985	(0.983)	0.017	(0.017)
Mean ± SD		1.828 ± 0.459	1.343 ± 0.568	(0.734 ± 0.238)	0.485 ± 0.426	(0.266 ± 0.238)

Abbreviations: AIS, American Spinal Injury Association Impairment Scale; IC, inspiratory capacity; V_RIP-rc_, respiratory inductance plethysmography volume of rib cage; V_RIP-ab_, respiratory inductance plethysmography volume of abdomen. Values in this table are expressed as mean ± SD.

## 4 Discussion

V_RIP_ and V_PN_ have been found to have a significant correlation (Fig. 1), and the measurement accuracy is considered to be high. Respiratory motion is caused by volume changes in two compartments, the thoracic and abdominal compartments, and the ventilation volume is expressed as the sum of the volume changes in the two compartments (Konno and Mead, 1967). RIP measures ventilation volume by calibrating the change in circumference that occurs in the thoracic and abdominal regions. The ventilation volume measured using a spirometer was used to calibrate the RIP's ventilation volume. Therefore, a significant correlation between V_RIP_ and V_PN_ is presumed, indicating high measurement accuracy. In addition, V_RIP_ places less stress on the subject because ventilation volume and breathing style can be evaluated simply by wearing a band around the rib cage and abdomen and performing breathing exercises. Furthermore, since RIP can measure the amount of change in the circumference of the thoracic and abdomen separately, the ventilation volume associated with breathing can be evaluated separately from the ventilation volume in the thorax and the ventilation volume in the abdomen. RIP is considered to be highly useful clinically.

**Fig. 2:**
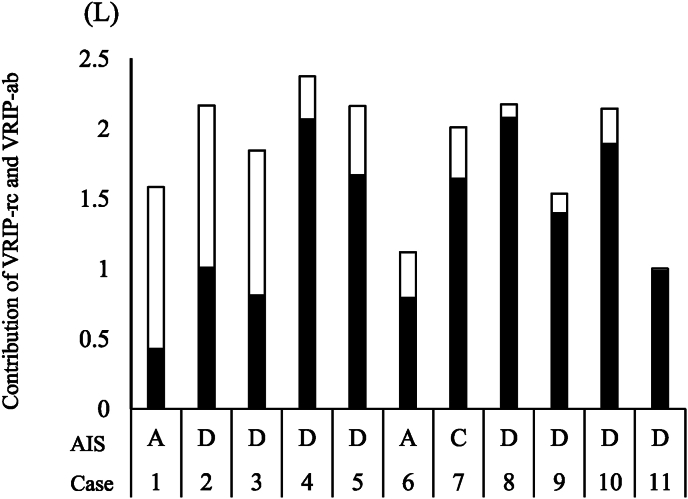
AIS and Contribution of V_RIP-rc_ and V_RIP-a_ to IC for Each Case. A closed bar chart shows V_RIP-rc_, and an open bar chart shows V_RIP-ab_. V_RIP-rc_ of AIS A cases was lower than that of AIS D cases Abbreviations: AIS, American Spinal Injury Association Impairment Scale; V_RIP-rc_, respiratory inductance plethysmography volume of rib cage; V_RIP-ab_, respiratory inductance plethysmography volume of abdomen; IC, inspiratory capacity.

This study found a significant correlation between V_RIP-rc_ and IC in patients with CSI. This result suggests that the better the function of the respiratory muscles that expand the rib cage, the greater the rib cage motion and the larger the IC. Lanza et al. (2013) investigated the relationship of rib cage motion during respiration to the respiratory muscle capacity and VC in healthy young adults. It has been reported that the PImax and VC were higher with greater rib cage motion, indicating that the functional status of the muscles of the rib cage affects respiratory function. Similar results were obtained in the present study, suggesting that rib cage motion may reflect the severity of CSI. For example, the respiratory muscles in AIS A cases may be less functional than those in AIS D cases. Therefore, it is considered that the V_RIP-rc_ of AIS A cases is lower than that of AIS D cases. Similarly, in the present study, the V_RIP-rc_ of Case 1 of AIS A was the lowest, and the V_RIP-rc_ of Case 8 of AIS D was the highest (Fig. 2, Table 4). This result supports that VRIP-rc reflects the severity of CSI and the ability of respiratory muscles to expand the rib cage.

**Fig. 3:**
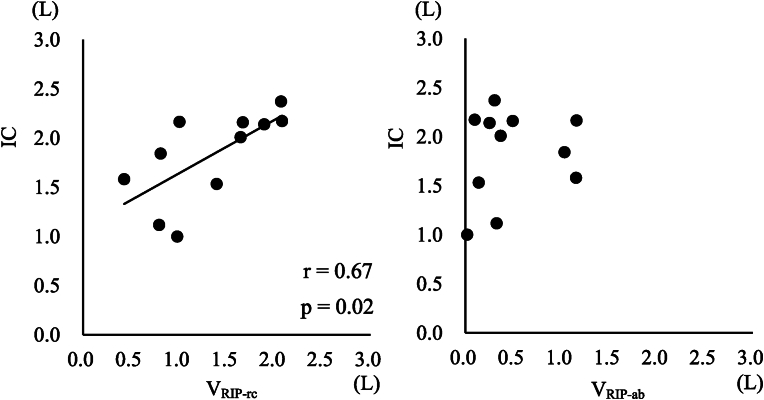
The relationship of IC to V_RIP-rc_ and V_RIP-ab_. IC and V_RIP-rc_ show a positive correlation, r = 0.67 (p = 0.02) (left figure). IC and V_RIP-ab_ show no correlation (right figure). Abbreviations: IC, inspiratory capacity; V_RIP-rc_, respiratory inductance plethysmography volume of the rib cage; V_RIP-ab_, respiratory inductance plethysmography volume of the abdomen.

On the other hand, V_RIP-ab_ and IC showed no correlation. V_RIP-ab_ indicates abdominal expansion resulting from diaphragmatic contraction and increases sufficiently in patients with CSI with no clear concurrent diaphragmatic paralysis, as in our research participants. Silver and Moulton (1969) reported that in some patients with cervical spinal cord injuries, the abdomen expands during inspiration but the thorax shrinks. This is attributed to the weakening of the muscles that stabilise the rib cage due to cervical spinal cord injury. Consequently, the rib cage is unable to resist the negative pressure created within the thoracic cavity by the diaphragm, leading to its inward pull. In other words, abdominal expansion may affect thoracic motion, which may lead to a decrease in IC. While no cases of thoracic shrinkage were observed in this study, it is possible that the expansion of the thorax was suppressed by contraction of the diaphragm. This suggest that V_RIP-ab_ and IC did not correlate.

Additionally, evaluating the motion of the thorax is considered suitable for evaluating recovery from paralysis. In a study using an animal model, Dougherty et al. (2012) evaluated the EMG of intercostal muscles after injury and reported a positive correlation between recovery of myoelectric potential and recovery of tidal volume. Since the intercostal muscles expand the rib cage, it is thought that the rib cage will also expand significantly when the intercostal muscles recover. In other words, assessing rib cage motion can also assess recovery from paralysis.

Based on the above, it is considered that rib cage motion is more suitable than abdominal motion for a simple assessment of respiratory function in patients with CSI.

This study had some limitations. It has been reported that thoracic mobility and respiratory function decrease with ageing. Participants in this study ranged in age from 30 to 80 years, and they were affected by an age-related decrease in thoracic mobility and respiratory function. However, the sample size of 11 participants was very small, and comparisons could not be made within the same age range. Therefore, it was impossible to exclude the effects of age-related decreases in thoracic mobility and respiratory function.

IC is influenced by rib cage motion in patients with CSI.

## Ethical approval

We certify that all applicable institutional and governmental regulations concerning the ethical use of human volunteers were followed during the course of this research. This study has been approved by the Ethics Committee of Ibaraki Prefectural University of Health Sciences (approval number: 830).

## Author contributions

RY was responsible for literature search, data collection, study design, analysis of data, manuscript preparation, and review of the manuscript.

KK was responsible for the analysis of the data and review of the manuscript.

YS was responsible for the analysis of the data and review of the manuscript.

HW was responsible for data collection, study design, and review of the manuscript.

NI was responsible for the study design and review of the manuscript.

MM was responsible for the study design and review of the manuscript.

HM was responsible for data collection, study design, and review of the manuscript.

KT was responsible for literature search, data collection, study design, analysis of data, manuscript preparation, and review of the manuscript.

## Funding

This research did not receive any specific grants from funding agencies in the public, commercial, or not-for-profit sectors.

## Declaration of competing interest

The authors have no competing interests in relation to the word described.

## Data Availability

Data will be made available on request.
